# Ultrasmall Superparamagnetic Magnetite Nanoparticles as Glutamate-Responsive Magnetic Resonance Sensors

**DOI:** 10.3390/s25144326

**Published:** 2025-07-10

**Authors:** Hannah Mettee, Aaron Asparin, Zulaikha Ali, Shi He, Xianzhi Li, Joshua Hall, Alexis Kim, Shuo Wu, Morgan J. Hawker, Masaki Uchida, He Wei

**Affiliations:** 1 Department of Chemistry and Biochemistry, California State University Fresno, 2555 E San Ramon Ave, Fresno, CA 93740, USA; hannahmettee@mail.fresnostate.edu (H.M.); ajasparin@mail.fresnostate.edu (A.A.); zulaikha28@mail.fresnostate.edu (Z.A.); xianzhi97@mail.fresnostate.edu (X.L.); joshuahall001@mail.fresnostate.edu (J.H.); alexis_kim@mail.fresnostate.edu (A.K.); morgan.hawker@mail.fresnostate.edu (M.J.H.); muchida@mail.fresnostate.edu (M.U.); 2 Department of Electrical and Computer Engineering, California State University Fresno, 2320 E San Ramon Ave, Fresno, CA 93740, USA; she3@mail.fresnostate.edu

**Keywords:** superparamagnetic iron oxide nanoparticles, eco-friendly, glutamate biosensors, magnetic resonance spectroscopy

## Abstract

Glutamate, the primary excitatory neurotransmitter in the central nervous system, plays a pivotal role in synaptic signaling, learning, and memory. Abnormal glutamate levels are implicated in various neurological disorders, including epilepsy, Alzheimer’s disease, and ischemic stroke. Despite the utility of magnetic resonance imaging (MRI) and magnetic resonance spectroscopy (MRS) in diagnosing such conditions, the development of effective glutamate-sensitive contrast agents remains a challenge. In this study, we present ultrasmall, citric acid-coated superparamagnetic iron oxide nanoparticles (CA-SPIONs) as highly selective and sensitive MRS probes for glutamate detection. These 5 nm magnetite CA-SPIONs exhibit a stable dispersion in physiological buffers and undergo aggregation in the presence of glutamate, significantly enhancing the T_2_ MRS contrast power. At physiological glutamate levels, the CA-SPIONs yielded a pronounced signal change ratio of nearly 60%, while showing a negligible response to other neurotransmitters such as GABA and dopamine. Computational simulations confirmed the mechanism of glutamate-mediated aggregation and its impact on transversal relaxation rates and relaxivities. The sensitivity and selectivity of CA-SPIONs underscore their potential as eco-friendly, iron-based alternatives for future neurological sensing applications targeting glutamatergic dysfunction.

## 1. Introduction

Glutamate is the primary excitatory neurotransmitter in the central nervous system, playing an essential role in synaptic transmission, learning, and memory. Abnormalities in glutamate signaling are associated with Alzheimer’s disease, ischemic stroke, schizophrenia, multiple sclerosis, chronic stress, and mood disorders [1,2,3,4]. For example, in patients with chronic stress, the release of glutamate has been found to malfunction in various brain regions [2]. Additionally, blood glutamate levels were demonstrated as significantly greater in patients with a major depressive disorder than in control groups [2]. In Alzheimer’s disease, the pathophysiology has been implicated by disturbances to the glutamatergic function. Considering that over 40% of the brain’s neuronal synapses are glutamatergic, these functions appear to play a significant role in Alzheimer’s disease [3]. Many such psychiatric disorders are strongly influenced by glutamatergic functions. Developing a practical biomedical tool for the in vivo monitoring of glutamate levels within the central nervous system would improve diagnostic abilities for neurodegenerative and neuropsychiatric conditions involving glutamatergic malfunctions. As such, this study aimed to develop a glutamate-sensitive magnetic resonance spectroscopy (MRS) tagging agent.

Glutamate is primarily found in the synaptic vesicles of neurons and glia of the brain and spinal cord [5]. Stored in the vesicles of nerve synaptic terminals, glutamate is released into the extracellular fluid, where its concentration rapidly increases. The balance of the glutamate concentration in extracellular fluid must be maintained [2]. The energy metabolism can be compromised when intracellular glutamate levels fall substantially below physiological norms (typically 8–10 millimolar, mM), as glutamate serves as a critical substrate in the tricarboxylic acid (TCA) cycle and plays a crucial role in maintaining the nitrogen balance [6]. On the contrary, excitotoxicity may arise when intracellular glutamate is elevated in combination with impaired glutamate transport or metabolism, which can lead to increased extracellular glutamate and the excessive activation of glutamate receptors, ultimately resulting in potential cell death [7,8]. As a result, a glutamate-sensitive tagging agent capable of detecting these subtle fluctuations would potentially serve as a valuable diagnostic tool.

Magnetic resonance imaging (MRI) is one of the most integral modern medical imaging techniques. It is especially valuable because it eliminates ionizing radiation, is minimally invasive, and provides an impressive resolution and contrast of soft tissues. Other medical imaging techniques, such as computed tomography (CT) scans or positron emission tomography (PET), use ionizing radiation or radioactive tracers [9,10]. To form an image, MRI aligns protons contained in water and fat tissues within the body to the strong magnetic field of a powerful magnet. A radiofrequency (RF) pulse is emitted, causing perturbations in the proton spin orientations and phase coherence. Following the RF pulse, protons return to their original spin orientation along the magnetic field [11]. Their return is detected by signal receivers within the MR machine. The time it takes for a proton to return to its spin orientation along the magnetic field is known as the longitudinal relaxation time, denoted as T_1._ The time it takes for a proton to lose phase coherence in the transverse plane is known as the transversal relaxation time, T_2_. Afterward, MRI scanners form images with different contrasts based on either the T_1_ and T_2_ times at each imaging pixel. As relaxation rates (R_1_ = 1/T_1_ or R_2_ = 1/T_2_) vary between tissues, MRI scanners construct contrasted images based on these differential R_1_ or R_2_, allowing a clear visualization between tissues. It is worth noting that magnetic resonance spectroscopy (MRS), a widely used spectroscopic technique, functions similarly to MRI and can provide important information, such as relaxation times, MR contrast power, and signal change amplitudes, about the images as MRI would form them. In this study, an MRS analysis was utilized to test the function of glutamate-sensitive T_2_ contrast agents.

**Figure 1:**
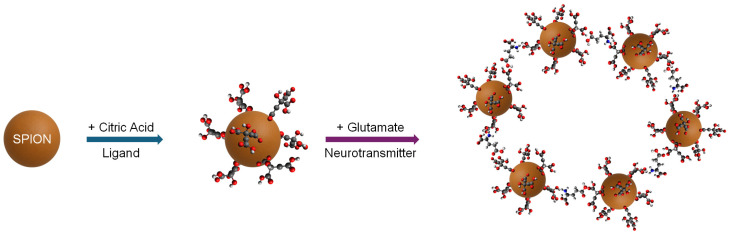
A schematic flowchart illustrates the coating of an SPION (brown sphere) with citric acid (CA) ligands to form CA-SPIONs and the subsequent aggregation of CA-SPIONs with glutamate molecules, which serve as crosslinkers. The carbon, oxygen, and nitrogen atoms are marked in gray, red, and blue, respectively.

Contrast agents are used to significantly enhance the contrast between target tissues or molecules and the surrounding tissues. Typically, on T_2_-weighted imaging, such as the sensors used in this study, cerebrospinal fluid and inflammation appear bright, whereas white matter appears dark gray [12]. Currently, the most used contrast agents in T_1_-weighted MR medical imaging are heavy metal gadolinium-based contrast agents (GBCAs). However, there have been concerns regarding their risk and accumulation in a small population of patients [13], such as seniors and those with impaired kidney function. For example, the administration of GBCAs is linked to nephrogenic systemic fibrosis (NSF), especially in patients with chronic kidney disease [14]. Additionally, evidence has demonstrated an association with GBCAs and gadolinium accumulation in tissues such as bone, the brain, and kidneys [15]. As such, there is an emerging demand for alternative contrast agents. In comparison with GBCAs, iron oxide nanoparticles have been demonstrated to have a much lower risk of causing NSF [16]. Considering that renal function typically declines with age and that elderly patients are more likely to undergo repeated imaging procedures [17], the development of heavy metal-free, biodegradable MRI contrast agents—such as CA-SPIONs—holds significant promise. Their iron-based composition, biocompatibility, and capacity for renal clearance further mitigate concerns related to long-term tissue accumulation. For this reason, superparamagnetic iron oxide nanoparticles (SPIONs) have received much attention [18] as MR contrast agents due to the eco-friendly nature of iron oxide, as well as their biocompatibility and stability [19]. SPIONs have been approved by many regions, such as the United States, Europe, and Japan, for use as magnetic resonance contrast agents in patients [20,21]. In our study, SPIONs were specifically developed to be glutamate-sensitive for their potential use as MRI contrast agents in the central nervous system. As displayed in Figure 1, ultrasmall 5 nm magnetite (Fe_3_O_4_) SPIONs were synthesized using a coprecipitation method adapted from the literature and our previous work [22,23,24,25,26]. Compared to other methods, coprecipitation as a method of synthesizing SPIONs is efficient and widely used, and it can be readily adopted for scale-up productions. Following the synthesis of SPION cores, various methods are available for stabilizing them in aqueous solutions [27]. The method in this study used citric acid (CA) to form citric acid-coated superparamagnetic iron oxide nanoparticles (CA-SPIONs), as the CA ligand’s tricarboxylic acid groups bind strongly to the iron oxide surface, and it has shown no toxicity to mammals [28]. Afterward, the physicochemical properties of CA-SPIONs were thoroughly characterized by multiple spectroscopic techniques, and the MR behaviors of CA-SPIONs dispersed in biological buffers were assessed using an MRS analysis. We hypothesized that the CA-SPIONs would aggregate in the presence of glutamate, due to the interaction between CA’s carboxylic acid groups and the amino group of glutamate. Therefore, the aggregated CA-SPIONs would increase the R_2_ relaxation rate and, in turn, enhance their T_2_ contrast power observable by MRS studies. This increment in the T_2_ contrast power would translate to the elevated hypointense contrast between the glutamate-rich area and the surrounding tissues. As a result, we have tested our 5 nm CA-SPIONs using MRS in the presence and absence of glutamate, dopamine, and gamma-aminobutyric acid (GABA) at their respective neurophysiological concentrations. Our results have successfully exhibited the sensitivity and specificity of these 5 nm CA-SPIONs as glutamate probes.

In the presence of glutamate, CA-SPIONs aggregate as depicted in Figure 1. Interactions between the citric acid (CA) ligands on our CA-SPIONs and the amino group of glutamate are facilitated by electrostatic attraction and hydrogen bonding. At a physiological pH, the negatively charged carboxylate groups of CA form selective interactions with the protonated amine groups of glutamate. This interaction is enhanced by the zwitterionic structure of glutamate and its proximity in terms of its size and charge density, which enables selective aggregation. In contrast, GABA and dopamine lack the same functional group arrangement, resulting in minimal or no aggregation.

## 2. Materials and Methods

### 2.1. Materials and Instrument

L-Glutamic acid monosodium salt, dopamine hydrochloride, ferrous chloride tetrahydrate (FeCl_2_·4H_2_O), ferric chloride hexahydrate (FeCl_3_·6H_2_O), iron standard (1000 μg/mL), sodium hydroxide (NaOH), hydrochloric acid (HCl), citric acid monohydrate, and bathophenanthroline (BPT) were purchased from Thermo Fisher Scientific (Waltham, MA, USA). GABA (γ-aminobutyric acid) was purchased from Sigma-Aldrich (St. Louis, MO, USA). All other chemicals were purchased from Thermo Fisher Scientific (Waltham, MA, USA) unless otherwise specified. Dynamic light scattering (DLS) analysis was performed in triplicate, with each measurement consisting of ten individual acquisitions, on a DynaPro NanoStar II system from Wyatt Technology (Santa Barbara, CA, USA). Magnetic resonance spectroscopy (MRS) measurements were obtained using a Bruker minispec MQ60 MR relaxometer from Bruker (Mannheim, Germany) operating at 1.5 Tesla and a Spinsolve 80 MR relaxometer from Magritek (Malvern, PA, USA) operating at 1.9 Tesla in these experiments. Fourier Transform Infrared (FT-IR) spectra were obtained using the Nicolet iS 10 FT-IR Spectrometer from Thermo Fisher Scientific (Waltham, MA, USA). UV-visible spectroscopy was carried out with a Cary 60 UV-Vis Spectrophotometer from Agilent (Santa Clara, CA, USA). A Talos F200C G2 Transmission Electron Microscope (TEM) from Thermo Fisher Scientific (Waltham, MA, USA) was used to determine the inorganic diameter of the synthesized magnetite SPIONs using our protocol described in Section 2.2. X-ray diffraction (XRD) was performed using a PANalytical X’Pert PRO Theta/Theta Powder X-ray Diffraction System from Malvern Panalytical (Malvern, United Kingdom), and X-ray photoelectron spectroscopy (XPS) relied on the use of a Thermo Fisher Scientific Nexus X-Ray Photoelectron Spectrometer (Waltham, MA, USA).

### 2.2. Synthesis of SPIONs

Magnetite superparamagnetic iron oxide nanoparticles (SPIONs) were synthesized from ferric chloride and ferrous chloride using a coprecipitation method in ambient conditions [22]. Ferrous chloride tetrahydrate (1.200 g) and ferric chloride hexahydrate (1.631 g) were combined in a 1:2 molar ratio in a beaker. Then, 5 milliliters of deionized water were added to this beaker, and the mixture was placed over tremendously vigorous magnetic stirring. Next, 5 milliliters of 8.50 M sodium hydroxide was added to the reaction mixture and allowed to react for 30 min at room temperature (RT) with continued vigorous stirring to ensure the uniform size of the formed SPIONs. The precipitated product was collected by vacuum filtration and washed five times with distilled water. The product was dried under a vacuum at room temperature overnight and then stored in a glass vial for future steps.

### 2.3. Coating SPIONs with Citrate Ligands

A 0.25 M citric acid (CA) solution was prepared using citric acid monohydrate (Waltham, MA, USA) and deionized (DI) water from California State University, Fresno (Fresno, CA, USA). To neutralize two of the triprotic hydrogens of citric acid and prevent the digestion of SPIONs, sodium hydroxide pellets were added to the solution until the CA solution’s pH reached 6.0. Over vigorous magnetic stirring and in a 90 °C oil bath, 5 milliliters of the citric acid solution was added to 100 mg of the SPIONs prepared in the previous step. This was left to undergo vigorous stirring for four hours, and the reaction mixture was stirred at RT until the SPIONs were homogeneously dispersed in the solution. The product solution was centrifuged, and its precipitate was discarded. The clear, brown supernatant was collected in a glass vial and stored for use.

### 2.4. Transmission Electron Microscopy

**Figure 2:**
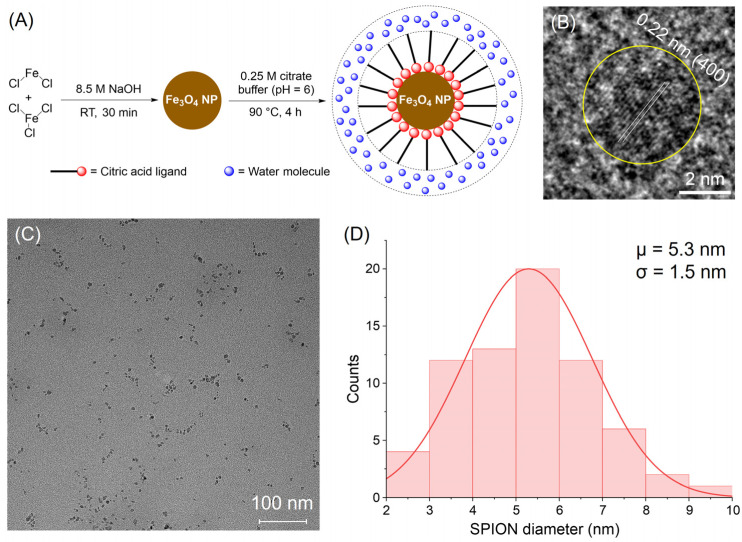
(**A**) The synthesis scheme for CA-SPIONs. (**B**) A high-resolution transmission electron microscopy (HR-TEM) image shows a single CA-SPION (circled in yellow) with lattice fringes. Scale bar: 2 nm. The measured interplanar distance of 0.22 nm corresponds to the interval of (400) planes in the magnetite. (**C**) A wide-view TEM image of CA-SPIONs. Scale bar: 100 nm. (**D**) A Gaussian size distribution of CA-SPIONs as determined by analyzing (**C**), demonstrating an average SPION inorganic core diameter of 5.3 ± 1.5 nm.

All TEM analyses were conducted using a Talos F200C G2 TEM machine (Thermo Fisher Scientific), operating at an accelerating voltage of 200 kV, with an image resolution of up to 0.1 nm, enabling the reliable visualization of lattice fringes and particle morphology. Samples of SPIONs were drop-cast onto a carbon-coated copper TEM grid and allowed to dry under vacuum prior to imaging. The wide-view TEM images (Figure 2C) were obtained from samples prepared at an approximate iron concentration of 0.3 mM. This moderate dilution minimizes artificial aggregation during sample drying while maintaining representative particle density for imaging. Nanoparticle size distribution was quantified from TEM images using ImageJ (Version 1.54p), with results shown as a histogram fitted to a Gaussian distribution.

### 2.5. Determining Iron Concentration of Citrate-Coated Sample Using UV-Vis

The following steps were employed to determine the exact concentration of iron in the citrate-coated sample. A Fe(NO_3_)_3_ iron standard of 1000 μg/mL [Fe^3+^], purchased from Thermo Fisher Scientific (Waltham, MA, USA) (CAS Number 7439-89-6) and certified (1000 μg/mL ± 5 μg/mL), was diluted to five standard samples of 5, 10, 20, 30, and 50 μg/mL (ppm). A 2.0 M acetate buffer with 10% *w/v* ascorbic acid (pH = 4.8) was prepared using sodium acetate and ascorbic acid. Hydrochloric acid and sodium hydroxide were used to adjust the pH. A 1 mg/mL solution of bathophenanthroline (BPT) was prepared as an indicator dye for the colorimetric determination of iron concentration.

The unknown sample was digested using a 1:1 ratio of the CA-SPIONs collected in Section 2.3 and 12.0 M concentrated hydrochloric acid. This sample was vortexed, then incubated in a water bath at 65 °C for 2 h. The digested sample was then diluted thirty times with DI water to bring the expected concentration within the range of iron standards (5–50 ppm). Then, the five iron standard solutions were separately mixed with the 2.0 M acetate buffer (containing 10% *w/v* ascorbic acid) and 1 mg/mL BPT solution. Each standard mixture was incubated for 20 min before being transferred to UV-Vis cuvettes for absorbance measurement at 540 nm. A calibration curve was obtained by linearly fitting the plot of absorbance values versus standard concentrations. Then, three parallel unknown digested CA-SPION samples were also mixed with the 2.0 M acetate buffer and 1 mg/mL BPT solution using the same ratios. Their triplicate concentrations were computed from the above calibration curve.

### 2.6. Characterization of CA-SPIONs with UV-Vis

UV-Vis absorption measurements were conducted between wavelengths of 300 and 800 nm. All measurements were obtained at room temperature using 1 cm path length quartz cuvettes. A blank spectrum of DI water was used for reference. Two aqueous samples containing CA-SPIONs were prepared at equivalent iron concentrations: the first contained only the CA-SPIONs, while the second contained CA-SPIONs and 10 mM glutamate. Each sample was sequentially measured via the Cary-60 UV-Vis spectrometer (Santa Clara, CA, USA).

### 2.7. Characterization of CA-SPIONs with FT-IR

Fourier Transform Infrared Spectroscopy (FT-IR) was performed for further characterization. Solid samples of uncoated SPIONs synthesized in Section 2.2 were analyzed without further preparation. CA-SPIONs were thoroughly dried in a vacuum desiccator at RT to obtain a solid powder for analysis. An additional citric acid monohydrate sample was also analyzed. The spectra of these three samples were obtained in the wavenumber range of 400 to 4000 cm^−1^, with 32 averaged scans and a 4 cm^−1^ resolution.

### 2.8. Characterization of SPIONs with XRD and XPS

X-ray diffraction (XRD) analyses were performed on the PANalytical X’Pert Pro diffractometer, where samples of the uncoated SPIONs were ground into a fine powder and used without any further modification. XPS measurements were performed using the Nexsa X-Ray Photoelectron Spectrometer with achromatic Al Kα radiation (1486.6 eV) and an incident beam oriented perpendicular to the sample surface normal. Both survey spectra (0–1300 eV), capturing photoemission signals from all constituent elements, and high-resolution scans of individual elemental regions were acquired using pass energies of 50 eV and 25 eV, respectively. High-resolution spectra were collected for C 1s and Fe 2p orbitals within their respective binding energy ranges, at 0.1 eV binding energy intervals, with a dwell time of 0.250 s. Signal averaging was performed over three scans for the survey spectra and five scans for the high-resolution regions. All the spectra were calibrated to the C 1s transition set at 285.00 eV [29]. Peak fitting was conducted using six curves with a mixed Gaussian–Lorentzian (30%:70%) curve shape [30] and a Shirley background to account for inelastic scattering contributions [31].

### 2.9. Testing Sensitivity Under MRS to Neurotransmitters:

The sensitivity of CA-SPIONs to differential central nervous system neurotransmitters, including glutamate, GABA, and dopamine, was tested using magnetic resonance spectroscopy (MRS) through a Bruker minispec MQ60 MR relaxometer operating at 1.41 (1.5) Tesla and through a Spinsolve 80 time-domain NMR with MR relaxometry function at 1.88 (1.9) Tesla, both at 37 °C. The measurements utilized a Carr–Purcell–Meiboom–Gill pulse sequence. For the T_2_ measurements on the Bruker relaxometer, repetition time T_R_ = 6000 milliseconds (ms) and echo time T_E_ = 30 ms with 200 echoes. For the T_2_ measurements on the Spinsolve relaxometer, repetition time T_R_ = 5000 milliseconds (ms) and echo time T_E_ = 250 ms with 20 echoes; for the T_1_ measurements on the same machine, repetition time T_R_ = 10,000 milliseconds (ms) and echo time T_E_ = 500 ms with 21 echoes. Afterward, the data obtained were fit to the T_2_-weighted MR signal equation S = k × exp[–T_E_/T_2_] or T_1_-weighted MR signal equation S = k × (1 − exp[–T_R_/T_1_]), where S is the amplitude of successive echoes of the MR signal, k is a constant of proportionality, T_1_ is the longitudinal relaxation time, and T_2_ is the transversal relaxation time. The goodness of fit of the data curves was consistently higher than 0.99. Then, transversal relaxivity (r_2_) values were calculated by linear fitting to graphs of relaxation rates (R_2_ = 1/T_2_) vs. CA-SPION concentration (c_Fe_), according to the equation R_2_ = 1/T_2_ = 1/T_2,0_ + r_2_⸳c_Fe_ = R_2,0_ + r_2_⸳c_Fe_, where T_2,0_ and R_2,0_ denote the solvent’s background (in the absence of CA-SPIONs, c_Fe_ = 0) transversal relaxation times and rates, respectively. The longitudinal relaxivity (r_1_) values were calculated in a similar manner.

To ensure that any observed sensitivity of CA-SPIONs under MRS to glutamate, GABA, or dopamine was appropriate at physiologically relevant levels, samples were prepared at previously reported physiological concentrations. The physiological intracellular concentrations of glutamate and GABA are typically 8–10 mM and 0.8–1.8 mM, respectively [32]. In contrast, the neurologically relevant intracellular dopamine concentration has yet to be decisively determined, and dopamine is predominantly found in extracellular compartments, with typical concentrations ranging from 0.25 to 2.5 μM [33,34]. To reflect this, we tested dopamine at both physiological (1.0 μM) and elevated concentrations (10.0 μM and 100.0 μM) to evaluate potential nonspecific aggregation. Consequently, the glutamate, GABA, and dopamine concentrations in our MRS studies were prepared at 10.0 mM, 1.0 mM, and 1.0–100.0 µM, respectively, and their pH values were adjusted to 7.0. Across this entire concentration range, CA-SPIONs exhibited no appreciable changes in relaxivity, reinforcing their specificity for glutamate. To determine the baseline r_2_ value of CA-SPIONs without any neurotransmitters, pure CA-SPIONs with five different clinically relevant iron concentrations (0.100, 0.233, 0.333, 0.499, and 0.665 mM) were prepared and analyzed using the Bruker minispec MR relaxometer in the MRS investigations. Their R_2_ relaxation rates were obtained by performing exponential fits on the MRS curves. Next, the R_2_ values were plotted against the known iron concentrations, followed by a linear fit that revealed their relationship: R_2_ = (5.311 ± 0.131 mM^−1^s^−1^)*⸳*c_Fe_ + (1.568 ± 0.054 s^−1^), with a satisfactory goodness of fit of R^2^ = 0.998. Thus, the baseline r_2_ of CA-SPIONs equals the slope, 5.311 ± 0.131 mM^−1^s^−1^; the background relaxation rate R_2,0_ is the intercept, 1.568 ± 0.054 s^−1^. Afterward, using the optimal iron concentration (0.333 mM), pure CA-SPION sensors (control) were first analyzed by the Bruker minispec MR relaxometer. Then, buffered solutions containing individual neurotransmitters at their physiological concentrations were mixed with the CA-SPION sensors (sample) before being analyzed by the Bruker minispec MR relaxometer as well. Each control or sample was separately prepared and measured in triplicate. Finally, their R_2_ numbers were obtained through fitting the MRS curves exponentially, and the corresponding r_2_ values for a single iron concentration were calculated using r_2_ = (R_2_ − R_2,0_)/c_Fe_, followed by the computation of r_2_ change ratios (r_2_%). Afterward, CA-SPIONs with varying glutamate concentrations (0–16.0 mM, near the physiological level) were also evaluated using Spinsolve 80 MR relaxometer (Malvern, PA, USA), and the results were analyzed in a similar manner.

### 2.10. Computational Interpretation of Glutamate-Sensitive CA-SPIONs

A MATLAB-based modeling system established in our recent work was utilized to measure and compare the transversal relaxation rates (R_2_) of protons induced by SPIONs [23]. MathWorks, Inc. (Natick, MA, USA) licensed this MATLAB software (Version R2025a) via California State University, Fresno, and the simulations were processed on our laboratory computing console (Nano Analytics II), consisting of 14 central processing unit (CPU) cores and 32 gigabytes of random-access memory (RAM). This system consisted of a central unit cell, adjacent to 3 × 3 × 3 identical unit cells, all of which consisted of protons. These protons, a component of simulated water molecules, were randomized in movement via a Monte Carlo algorithm within the periodic boundary conditions. Measurements of the magnetic perturbations induced by the SPIONs were then calculated for every single unit cell. The simulation model was designed to isolate the effects of SPION aggregation on proton relaxation, utilizing a periodic unit cell with randomly positioned water proton sites. While this abstraction captures key magnetic field perturbations and Monte Carlo-based diffusion effects, it does not fully account for the complexity of intracellular environments, such as spatial crowding, nonspecific binding, and dynamic viscosity. Additionally, protein corona effects, pH gradients, or competitive ion interactions were not included. These simplifications facilitate computational tractability but represent limitations when translating results to in vivo settings. Future improvements will incorporate biological macromolecule modeling and heterogeneous proton density distributions.

To align with common T_2_-w MRI conditions [35,36,37], certain specifications regarding material and MR pulse sequence parameters were standardized throughout the simulation process, as developed and described in our recent work [23]. All SPION diameters were kept at 10 nm, which is slightly larger than the 5 nm inorganic diameter observed by TEM but ensures a 4 times faster simulation process and, in turn, can evaluate more SPION aggregation configurations under the same time frame [38]. The simulation unit cell is a cube, with the three dimensions a = b = c = 1000 nm, exactly 100 times the nanoparticle diameter. Under this setting, the iron concentration of C_Fe_ is maintained at 0.21 mM, close to the in vitro C_Fe_ of 0.100–0.665 mM used in MRS studies. This C_Fe_ value compares reasonably well with the iron dosage in typical MRI and MRS scans. In addition, the T_E_ values in the simulation (up to 10 ms) were selected for computational efficiency and do not aim to directly replicate the experimental T_E_ values, which are three times longer and would otherwise require a triple simulation time. While absolute R_2_ values may differ, the percent change (ΔR_2_%) between aggregated and disaggregated states remains valid for comparison, as it reflects the relative enhancement due to aggregation. In the end, the normalized MR signal intensities and T_E_ points were then plotted on a figure, with a maximum echo time T_E_ of 10 ms and fifty T_E_ points at an interval of 0.2 ms being used for the linear fitting—the slope of which being equivalent to the transversal relaxation rates R_2_ and representative of the observable T_2_ contrast power of the SPION for MRI.

The glutamate sensor manifests in two states during the sensing and simulation process. A state corresponding to a higher neurochemical concentration of glutamate is defined as “On,” while a state of lower neurochemical concentration is defined as “Off,” performing as described. These On and Off states were experimentally controlled by the aggregation or disaggregation of SPIONs, respectively. Performance of the sensor was then measured by the signal change ratio, defined as $∆R_{2}\%=\frac{\left(R_{2,On}-R_{2,Off}\right)}{R_{2,Off}}\times 100\%$. The higher the signal change ratio is, the more preferable it is in terms of better MRI sensor performance and sensor configuration.

For the On state of the glutamate sensor, citric acid (CA) ligands stabilize each SPION, allowing glutamate to crosslink two adjacent CA-SPIONs in a high glutamate concentration environment. Five different plausible configurations for the glutamate sensor were then proposed and simulated for the On state of the glutamate sensor: a dimer, trimer, tetramer, pentamer, and hexamer, named according to the number of SPIONs in each sensor configuration. For the Off state, a lack of glutamate presence leads to the free dispersion of CA-SPIONs in the biological environment. For both On and Off state simulations, 25 randomized SPION cluster positions, with 100 randomized protons from water molecules, were computed, ensuring statistically reliable results with constant fitting goodness over 0.99. These R_2,On_ and R_2,Off_ results were collected and simulated in triplicate.

## 3. Results and Discussion

### 3.1. TEM and Synthesis Scheme

The synthesis of citric acid-coated superparamagnetic iron oxide nanoparticles (CA-SPIONs) was carried out via a two-step process, as illustrated in Figure 2A. First, SPIONs were generated by coprecipitating ferrous and ferric chloride salts in a 1:2 molar ratio using 8.5 M NaOH at room temperature for 30 min. These SPIONs were then coated with 0.25 M citric acid at 90 °C for four hours, yielding water-dispersible CA-SPIONs. This surface functionalization with citric acid improves the colloidal stability and introduces carboxylate groups, enabling a future conjugation or sensing functionality. Next, the high-resolution transmission electron microscopy (HR-TEM) analysis, shown in Figure 2B, reveals lattice fringes with a measured interplanar distance of 0.22 nm for a single nanoparticle (circled in yellow). This spacing matches the (400) crystal planes of the magnetite (Fe_3_O_4_). Together with the following X-ray investigations, this suggests that the synthesized nanoparticles comprise magnetite. Such crystallographic information affirms the synthesized material’s structural identity and magnetic integrity, which are essential for ensuring reproducible magnetic behavior in imaging applications. In parallel, a broader field TEM view (Figure 2C) shows that the CA-SPIONs are well-dispersed, with no significant aggregation. This dispersion supports the effectiveness of the citric acid coating in preventing interparticle agglomeration, a crucial property for both colloidal stability and sensing applications.

The further statistical analysis of the particle diameters from the wide-field image is presented in Figure 2D, demonstrating a Gaussian size distribution with an average inorganic core diameter of 5.3 ± 1.5 nm. This places the nanoparticles in the ultrasmall category (<10 nm), which is particularly desirable for biomedical applications. Ultrasmall SPIONs are known to possess extended blood circulation times, an improved MR relaxivity due to a high surface-area-to-volume ratio, and improved renal clearance [24], which reduces the post-diagnosis iron exposure and enhances their safety profile. The successful synthesis of ultrasmall CA-SPIONs with a well-defined magnetite structure and uniform size underscores their potential for sensitive magnetic resonance imaging (MRI) and magnetic resonance spectroscopy (MRS) applications [39].

### 3.2. X-Ray Diffraction and X-Ray Photoelectron Spectroscopy

**Figure 3:**
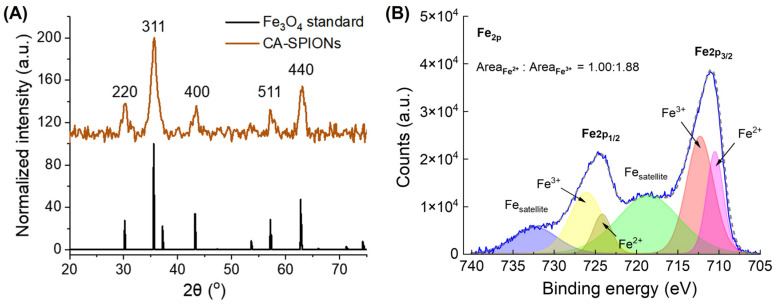
(**A**) The X-ray diffraction of 5 nm magnetite SPIONs, where the *x*-axis is the diffraction angle (2θ) in degrees and the *y*-axis is signal counts. The diffraction pattern displays prominent peaks at 30.4°, 35.6°, 43.4°, 57.4°, and 62.7°, which correspond to the (220), (311), (400), (422), (511), and (440) crystal planes, respectively. (**B**) X-ray photoelectron spectroscopy of 5 nm magnetite SPIONs, where the *x*-axis is the binding energy in eV and the *y*-axis is signal counts. The solid blue line and dashed gray line represent the experimental data and the summation of the six fitted curves, respectively. The ratio of Fe^2+^ to Fe^3+^ peak areas in the SPIONs was determined to be 1:1.88, which is close to 1:2 and consistent with the stoichiometry for FeO·Fe_2_O_3_.

The crystallinity of the as-synthesized magnetite (Fe_3_O_4_) SPIONs was confirmed by the X-ray diffraction (XRD) analysis, as presented in Figure 3A. The diffraction pattern displays prominent peaks at 30.4°, 35.6°, 43.4°, 57.4°, and 62.7°, which correspond to the (220), (311), (400), (422), (511), and (440) crystal planes, respectively [40]. These reflections are characteristic of the inverse spinel structure of Fe_3_O_4_ (space group F-43m), which is consistent with the standard JCPDS card No. 19-0629 [41]. The broadening of the diffraction peaks is attributed to lattice distortion and size confinement effects associated with the nanoscale dimensions of the Fe_3_O_4_ SPIONs [42]. As a result, this crystalline profile supports the synthesized nanoparticles’ high phase purity and uniformity, which is essential for reproducible magnetic behavior and a consistent MR performance. To gain a more comprehensive understanding of the surface electronic states of Fe_3_O_4_ nanoparticles, X-ray photoelectron spectroscopy (XPS) was performed. As illustrated in Figure 2A, the Fe 2p high-resolution spectrum of the SPIONs exhibits characteristic peaks at 711.0 eV (Fe 2p_3/2_) and 724.6 eV (Fe 2p_1/2_) [43]. The deconvolution of the Fe 2p_3/2_ peak confirmed the mixed-valence nature of iron in Fe_3_O_4_ [30,44], with an Fe^2+^ to Fe^3+^ ratio of approximately 1:1.88, which is in close agreement with the theoretical stoichiometry of 1:2. The presence of both oxidation states ensures the magnetite structure, which has an improved magnetic susceptibility and superparamagnetic behavior than other phases of iron oxide [45], contributing to the favorable T_2_ contrast properties observed in the CA-SPIONs.

### 3.3. UV-Vis and IR

**Figure 4:**
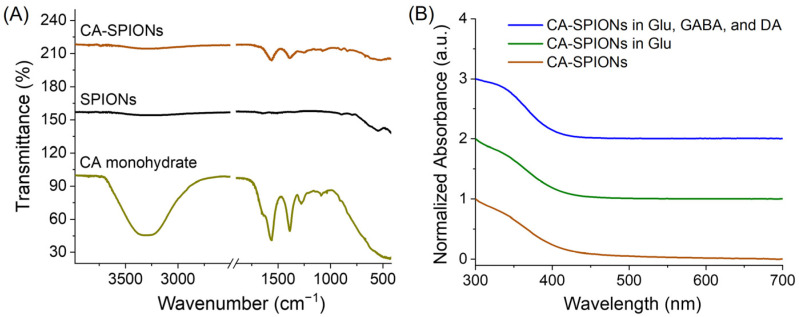
(**A**) Fourier Transform Infrared (FT-IR) spectra of the CA-SPIONs (brown), uncoated SPIONs (dark), and citric acid (CA) monohydrate (yellow-green), where the *x*-axis is the wavenumber (cm^−1^) and the *y*-axis is the transmittance (%). In the brown curve, the dual characteristic CA peaks around 1500 cm^−1^ confirmed the successful coating of CA onto SPIONs. (**B**) UV-Vis spectra. Brown: CA-SPIONs alone; green: CA-SPIONs in 10 mM glutamate (Glu); blue: CA-SPIONs in 10 mM glu, 1 mM GABA, and 1 μM dopamine (DA) dispersed in phosphate-buffered saline (PBS) 1X. The *x*-axis is the wavelength (nm), and the *y*-axis is the normalized absorbance (a.u.). The similarity between CA-SPIONs with and without glutamate ruled out biofouling and demonstrated the colloidal stability of our SPION sensor at the physiological intracellular concentration of 10 mM glutamate.

As shown in Figure 4A, the FT-IR spectrum of uncoated SPIONs (dark curve) displayed a prominent absorption region near 540 cm^−1^, which is attributed to the Fe–O stretching vibration characteristic of magnetite nanoparticles [46]. The IR spectrum of citric acid (CA) showed a broad peak around 3250 cm^−1^ due to O–H stretching from intermolecularly hydrogen-bonded water molecules and two distinct peaks at ~1575 cm^−1^ and ~1400 cm^−1^, corresponding to asymmetric and symmetric stretching vibrations of carboxylate (COO^−^) groups, respectively [47]. In addition, the C=O shoulder at ~1700 cm^−1^, characteristic of the free acid form of citric acid, is present in the spectrum of citric acid monohydrate (yellow-green curve in Figure 4A). Notably, the C=O shoulder near 1700 cm^−1^ is absent in the CA-SPIONs spectrum (brown curve in Figure 4A), indicating the lack of substantial free or unbound citric acid and suggesting a net negative zeta potential of CA-SPIONs. Moreover, the COO^−^ peaks were retained at ~1575 cm^−1^ and ~1400 cm^−1^ in the CA-SPIONs spectrum, confirming the presence of the surface-bound citric acid and successful ligand exchange during the coating process. This indicates that citric acid molecules were effectively anchored onto the nanoparticle surfaces through the coordination between carboxylate groups and iron ions, thereby enhancing the solubility and colloidal stability. Moreover, the IR data verify surface functionalization and suggest an improved surface chemistry for biological dispersion. The UV-Vis spectrum of CA-SPIONs (Figure 4B) exhibited a broad, featureless absorption extending from the visible into the near-infrared region, consistent with the spectral profile of magnetite nanoparticles due to electronic transitions within the Fe^2+^ and Fe^3+^ sublattices [48]. This optical response conforms to the identity of the iron oxide core. Furthermore, the nearly identical absorbance spectra of CA-SPIONs under three different neurotransmitter conditions (no neurotransmitter, 10 mM glutamate, as well as a mixture of 10 mM glutamate, 1 mM GABA, and 1 μM dopamine dispersed in PBS 1X) rule out undesired particle aggregation under physiological intracellular conditions, an otherwise common biofouling concern in nanoparticle-based biosensors [49]. As a result, this UV-Vis spectral stability affirms the colloidal robustness of the citrate coating, which maintains a satisfactory nanoparticle dispersion even in a neurochemical-rich environment. Prior studies employing citric acid ligands for coating SPIONs have established citric acid as a stable and robust ligand on SPIONs [50]. Additionally, the previous literature reports zeta potential values of CA-SPIONs in the range of −30 to −50 mV, indicating an excellent colloidal stability [50,51,52].These zeta potential values from the literature suggest strong electrostatic repulsion forces between CA-SPIONs in physiological conditions, thereby increasing their colloidal stability, which is to be confirmed with additional hydrodynamic measurements in the following studies.

### 3.4. Glutamate Sensing Through MRS Studies

It is expected that the aggregation of CA-SPIONs (On state) to any neurotransmitter would decrease the T_2_ relaxation time from the baseline T_2_ relaxation time of the disaggregated CA-SPIONs (Off state) in the absence of that neurotransmitter. By comparing the aggregated T_2_ relaxation time to the disaggregated T_2_ relaxation time, the T_2_ and R_2_ signal change ratio can be determined. Then, higher signal change ratios indicate a stronger sensitivity and an elevated contrast change in MRI and MRS. Measuring the T_2_ relaxation times of the samples containing CA-SPIONs in the presence of each neurotransmitter at physiological concentrations and the samples of the CA-SPIONs alone could allow the determination of the CA-SPION sensitivity to each neurotransmitter through comparative signal change ratios.

**Figure 5:**
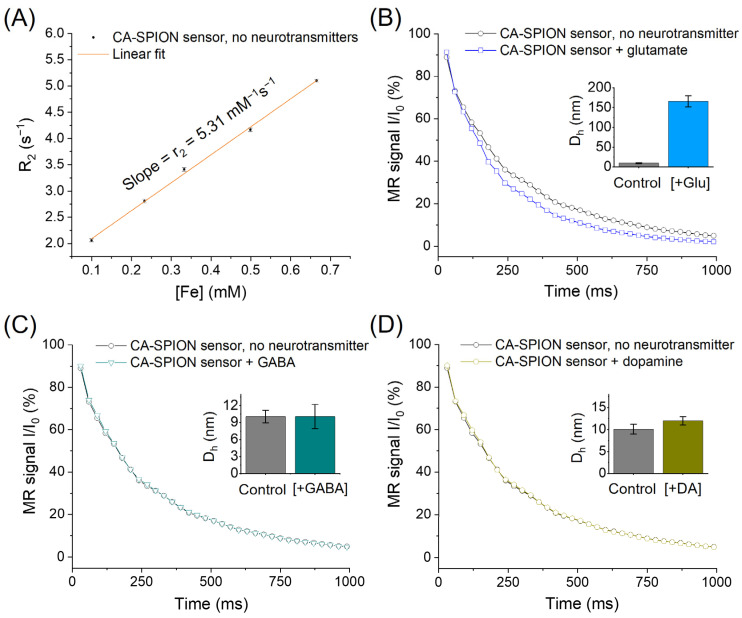
(**A**) MRS of CA-SPION sensors at 1.5 Tesla in the absence of neurotransmitters, in which the *x*-axis represents the iron concentrations of CA-SPION samples in mM and the *y*-axis represents the R_2_ relaxation rates in s^−1^. The linear fit model yields a baseline r_2_ value of 5.31 mM^−1^s^−1^ at 1.5 Tesla. (**B**) MRS of CA-SPION sensors in the presence of glutamate (blue) vs. the absence of glutamate (dark), in which the *x*-axis is the time in ms and the *y*-axis is the relative MR signal intensity. (**C**) MRS of CA-SPIONs in the presence of GABA (teal) and the absence of GABA (dark). (**D**) MRS of CA-SPIONs in the presence of dopamine (yellow) and the absence of dopamine (dark). Insets: The hydrodynamic diameter (D_h_) of the CA-SPION sensor without and with neurotransmitters (Glu: glutamate, GABA: γ-aminobutyric acid, and DA: dopamine), as determined by three parallel dynamic light scattering measurements. The error bars indicate the standard error of the mean.

Figure 5A shows the transversal relaxivity (r_2_) of CA-SPIONs measured by magnetic resonance spectroscopy (MRS) without neurotransmitters. The linear fit of the R_2_ relaxation rates as a function of the iron concentration yields an r_2_ value of 5.31 mM^−1^s^−1^, which is consistent with other ultrasmall, well-dispersed SPIONs reported in the literature [53]. This moderate r_2_ relaxivity reflects an efficient spin–spin relaxation enhancement, suggesting that the CA-SPIONs possess a good colloidal stability and a narrow size distribution. Such monodispersity is favorable for predictable behavior in biological media, offering advantages such as a prolonged circulation time, reduced aggregation, and consistent imaging contrast [54]. Figure 5B compares the MRS signal decay of CA-SPIONs in the presence (blue) and absence (dark) of 10.0 mM glutamate, a physiologically relevant concentration. A faster decay in the signal intensity is observed with glutamate, indicating a shorter T_2_ relaxation time (i.e., increased R_2_ value). This result demonstrates that CA-SPIONs exhibit a sensitivity to glutamate, with an enhanced contrast power under MRS conditions. The interaction between the surface CA ligands and glutamate presumably facilitates localized proton dephasing near the SPION surface, accounting for the observed change in the relaxation behavior. Dynamic light scattering (DLS) measurements (Figure 5B inset) revealed that the CA-SPION sensor had an average hydrodynamic diameter (D_h_) of 10.1 ± 1.1 nm in the absence of neurotransmitters. In contrast, the CA-SPION sensor with 10.0 mM glutamate demonstrated an average diameter of 165.7 ± 14.2 nm, approximately 16 times its original size (detailed size distribution curves are shown in Figure S1). This observation validates the aggregation-based sensing mechanism of our CA-SPION probe.

Figure 5C evaluates the response of CA-SPIONs to the 1.0 mM GABA, while Figure 5D examines the response to the 1.0 µM dopamine, both within physiologically relevant ranges. In both cases, the signal decay curves in these neurotransmitters’ presence (teal or yellow) and absence (dark) are virtually indistinguishable. Moreover, the DLS results (Figure 5C,D insets) showed that the D_h_ of the CA-SPION in the 1.0 mM GABA and 1.0 µM dopamine is 10.1 ± 2.1 and 12.0 ± 0.9 nm, respectively, both of which are close to the original size of the CA-SPION without neurotransmitters (10.1 ± 1.1 nm). The CA-SPIONs were also evaluated at higher dopamine concentrations (10.0 and 100.0 µM), again showing no significant change in the relaxation rate compared with the control (Figure S2 and Table S1). This consistent lack of a response confirms that the CA-SPIONs exhibit a selectivity for glutamate over GABA and dopamine, which are essential for developing neurotransmitter-specific contrast agents in neuroimaging. These findings underscore the potential of CA-SPIONs as sensitive and selective sensors for glutamate in magnetic resonance-based modalities. Such a selective responsiveness and stable relaxometric performance position these nanoparticles as strong candidates for future development in functional MRI/MRS and neurodiagnostic applications [55].

**Figure 6:**
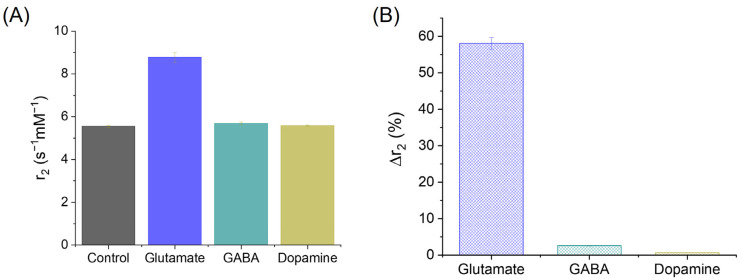
(**A**) Transversal relaxivities of CA-SPIONs in the control (neurotransmitter-absent), glutamate, GABA, and dopamine samples, where the *x*-axis represents the different CA-SPION samples, and the *y*-axis represents the relaxivity values (r_2_ in s^−1^mM^−1^). (**B**) The r_2_ change ratios of CA-SPIONs in the presence of glutamate, GABA, and dopamine, when compared to the control samples lacking neurotransmitters, where the *x*-axis is the different neurotransmitters, and the *y*-axis is the transversal relaxivity change ratio expressed as a percentage (Δr_2_%).

Figure 6A presents the measured transverse relaxivity (r_2_) values of CA-SPIONs under four conditions: the control (no neurotransmitter) and in the presence of glutamate, GABA, and dopamine. The respective r_2_ values are 5.55 ± 0.06, 8.77 ± 0.24, 5.69 ± 0.07, and 5.58 ± 0.03 s^−1^mM^−1^. While the r_2_ values remain relatively unchanged between the control, GABA, and dopamine conditions, a substantial increase was observed in the presence of glutamate. This enhancement in the r_2_ suggests that the CA-SPIONs exhibit a pronounced sensitivity to glutamate, with minimal interference from other neurotransmitters, such as GABA and dopamine, at physiologically relevant concentrations. Figure 6B quantifies this observation by illustrating the relative change in relaxivity (Δr_2_%) compared to the control. The calculated Δr_2_% values are 58.1 ± 1.6% for glutamate, 2.6 ± 0.1% for GABA, and 0.64 ± 0.01% for dopamine. A ~60% increase in the r_2_ relaxivity in response to glutamate indicates a robust sensitivity of the CA-SPIONs compared to other reported MRI probes [56,57]. In contrast, the negligible changes in relaxivity in response to GABA and dopamine demonstrate the selectivity of the CA-SPION system for glutamate detection. These results demonstrate that CA-SPIONs offer a sensitive and selective MR contrast enhancement in the presence of glutamate, while maintaining a stable relaxometric performance in the presence of other neurotransmitters. This specificity highlights the potential of CA-SPIONs as responsive probes for in vitro glutamate sensing, particularly in neuron-derived cell lines or other neurochemical assays. Given the central role of glutamate in excitatory neurotransmission and numerous neuropsychiatric disorders, the ability to reliably detect glutamate via non-invasive magnetic techniques could potentially contribute to future diagnostic approaches in neuroscience and neuroimaging. In comparison, while several glutamate-sensing strategies have been developed, such as aptamer-based fluorescent probes, enzyme-functionalized electrochemical sensors, and polymeric nanocomposites, these platforms often require complex modification steps to achieve the target specificity [58,59,60]. Notably, our CA-SPION platform exhibits an excellent colloidal stability, selective glutamate-induced aggregation, and strong T_2_ contrast enhancement, all without requiring enzymatic or biological recognition elements. Moreover, it is worth noting that glutamate-weighted chemical exchange saturation transfer (GluCEST) is an innovative method of indirectly observing the relative concentrations and spatial distribution of glutamate in the human brain. The GluCEST method primarily operates within the millimolar range, which is comparable to our findings in this study. In general, GluCEST requires a magnetic field strength exceeding 7 Tesla, which is typically available in advanced research and development settings [61]. In contrast, our measurements were conducted at a significantly lower field strength of approximately 1.5 Tesla, aligning with the operational range of standard clinical MRI scanners, which commonly function between 1.5 and 3.0 Tesla.

**Figure 7:**
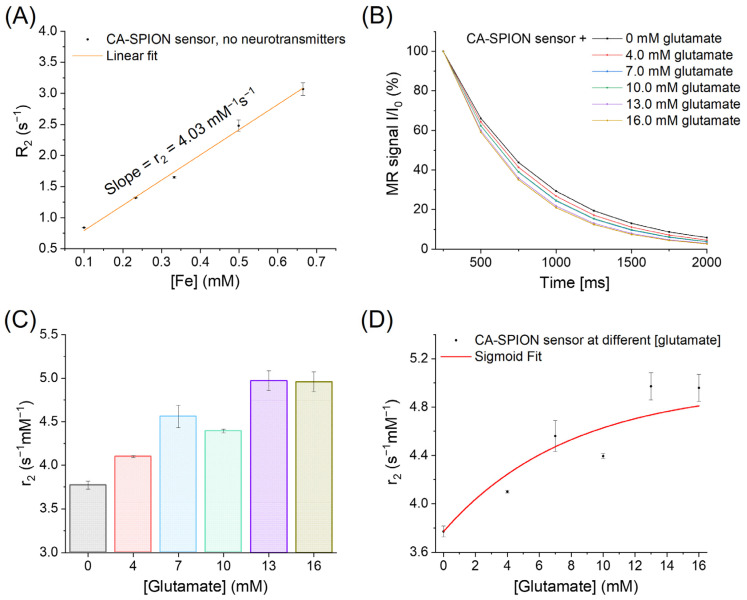
(**A**) MRS of CA-SPION sensors at 1.9 Tesla in the absence of neurotransmitters, in which the *x*-axis represents the iron concentrations of CA-SPION samples in mM, and the *y*-axis represents the R_2_ relaxation rates in s^−1^. The linear fit model yields a baseline r_2_ value of 4.03 mM^−1^s^−1^ at 1.9 Tesla. (**B**) MRS of CA-SPION sensors at varied glutamate concentrations: 0 (dark), 4.0 (red), 7.0 (light blue), 10.0 (light green), 13.0 (purple), and 16.0 (yellow-green) mM. The *x*-axis is time in ms, and the *y*-axis is the relative MR signal intensity. (**C**) Transversal relaxivities of CA-SPIONs in different glutamate concentrations ranging from 0 to 16.0 mM, where the *x*-axis represents the different glutamate concentrations, and the *y*-axis represents the relaxivity values (r_2_ in s^−1^mM^−1^). (**D**) The sigmoid fit of the transversal relaxivities of CA-SPIONs at the specified glutamate concentration range, which demonstrates a saturable trend of the r_2_ values.

To further elucidate the glutamate-responsive behavior of CA-SPIONs near physiological intracellular concentrations, we performed MRS measurements across a glutamate gradient ranging from 0 to 16.0 mM. As shown in Figure 7A, baseline measurements at 0 mM glutamate yielded a transversal relaxivity (r_2_) value of 4.03 mM^−1^s^−1^ at 1.9 Tesla. Serial additions of glutamate (4.0, 7.0, 10.0, 13.0, and 16.0 mM) led to a steady acceleration in the MR signal decay, as seen in Figure 7B, indicating shorter T_2_ relaxation times with increasing glutamate concentrations. This trend supports a dose-dependent enhancement of R_2_, which is consistent with the glutamate-induced aggregation of CA-SPIONs. The calculated r_2_ values for each concentration are further presented in Figure 7C, demonstrating a sigmoidal increase in relaxivity: from 3.77 ± 0.05 mM^−1^s^−1^ at 0 mM glutamate to a peak of 4.97 ± 0.11 mM^−1^s^−1^ at 13.0 mM, after which it plateaued (4.96 ± 0.11 mM^−1^s^−1^ at 16.0 mM). This saturable trend, fitted with a sigmoid model in Figure 7D, suggests that beyond a threshold glutamate concentration (13.0 mM), aggregation-induced changes in relaxivity approach their upper limit, likely due to the maximal interparticle crosslinking mediated by glutamate molecules. Furthermore, the CA-SPION probe demonstrates an r_2_ increase starting at approximately 4.0 mM glutamate and plateauing near 13.0 mM. This range aligns with normal intracellular glutamate concentrations (8–10 mM) but also suggests that pathologically elevated levels—such as those seen during compromised energy metabolism (less than 5 mM glutamate) or excitotoxic events (higher than 10 mM glutamate)—could result in a pronounced contrast enhancement. Therefore, by calibrating signal thresholds and incorporating r_2_ quantification protocols, our sensor could help differentiate typical physiological concentrations from abnormally high glutamate levels associated with neurodegeneration or metabolic dysfunction.

While this study demonstrates a robust glutamate response from CA-SPIONs in physiologically relevant concentration ranges (0–16 mM), further work is needed to quantify the absolute detection limit under complex biological conditions. Based on the sigmoidal relaxivity trend observed (Figure 7D), a detectable r_2_ increase begins at approximately 4.0 mM glutamate. The midpoint of the transition occurs near 10.0 mM, which corresponds well with intracellular glutamate concentrations in neurons and glial cells. However, extracellular glutamate concentrations—particularly in cerebrospinal fluid (CSF) or serum—are typically in the micromolar range. Future developments will therefore aim to increase the sensor’s sensitivity and signal resolution to enable the detection in dilute environments. Additionally, applications in real biological fluids will require an evaluation of matrix effects such as the protein corona formation and nonspecific interactions, which could affect the colloidal stability and aggregation behavior. Ongoing studies are focused on surface functionalization strategies to preserve selectivity and enhance biocompatibility in serum and CSF-like media.

To assess whether longitudinal relaxivity (r_1_) might also reflect glutamate sensitivity, we conducted parallel T_1_-weighted MRS experiments over the same glutamate concentration range, as well as for GABA and dopamine (Figure S3 and Table S2). However, r_1_ values remained mostly unchanged—showing fluctuations generally under 10% without a discernible trend—indicating that the CA-SPION aggregation exerts a minimal influence on the T_1_ contrast. This observation aligns with the established knowledge that the SPION-induced r_2_ modulation is predominantly governed by spatial clustering, while r_1_ is more sensitive to surface proton exchange dynamics and the coordination environment, which remain largely unaffected by interparticle aggregation [19]. These findings highlight the r_2_-selective and concentration-dependent behavior of CA-SPIONs for glutamate sensing in the intracellular physiological range. The observed sigmoid profile not only mimics biological signaling thresholds but also offers a quantifiable readout for monitoring glutamatergic fluctuations with a high dynamic sensitivity. The ability to operate effectively at clinically relevant magnetic field strengths further underscores the translational promise of CA-SPIONs, as it aligns with the capabilities of widely deployed MRI systems in medical practice.

### 3.5. MATLAB-Based Computational Simulations

**Figure 8:**
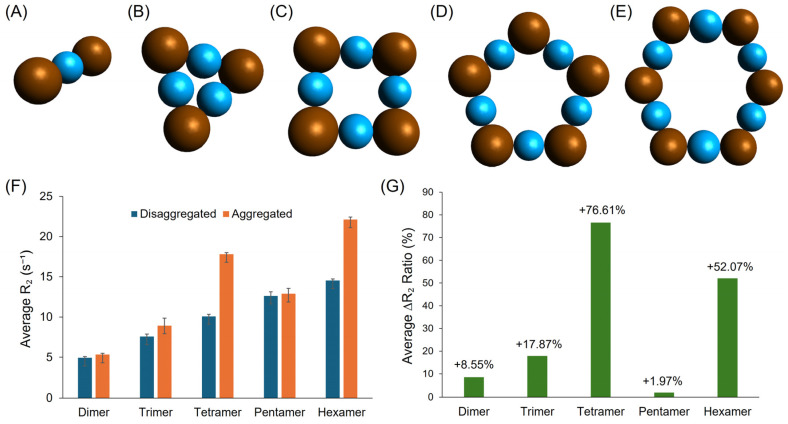
Five proposed stimulus-responsive aggregation (On state) models for the CA-SPION-based glutamate sensor: the (**A**) dimer, (**B**) trimer, (**C**) tetramer, (**D**) pentamer, and (**E**) hexamer. The SPIONs are shown as brown spheres, while the glutamate linkers are shown as light blue spheres. (**F**) A side-by-side comparison of the average transversal relaxation rate (R_2_) results of the aggregated (orange, On state) vs. disaggregated (dark blue, Off state) CA-SPION sensors gathered from simulations for five proposed aggregation models. The error bars represent the standard deviations of the triplicate simulations. (**G**) The percent change values from disaggregated to aggregated states for all five models.

To elucidate the underlying mechanism of CA-SPION-based glutamate sensing, five stimulus-responsive aggregation models were proposed and simulated in MATLAB, representing possible “On” states of the sensor: the dimer, trimer, tetramer, pentamer, and hexamer. These configuration models are depicted in Figure 8A–E. Each model was analyzed in both aggregated (“On”) and disaggregated (“Off”) states, and simulations were repeated in triplicate to ensure statistical robustness (R^2^ > 0.99 for all fits). The “Off” state corresponds to baseline glutamate concentrations near or below 4 mM, which are below physiological intracellular levels. The “On” state arises from the increasing aggregation in the range of 8–10 mM. Pathological elevations over 10 mM—such as those observed in ischemic stroke or neurodegeneration—may exceed this range transiently. The transversal relaxation rate (R_2_) values for all five aggregated configurations were consistently elevated compared to their disaggregated counterparts, as shown in Figure 8F. This trend indicates that the formation of SPION aggregates via glutamate-mediated crosslinking leads to enhanced magnetic susceptibility effects, resulting in a faster T_2_ relaxation and an increased T_2_ contrast power. Notably, R_2_ increased gradually with higher-order aggregates, culminating in the hexamer model, which exhibited the highest average R_2_ value of 22.1 ± 0.3 s^−1^. Furthermore, Figure 8G summarizes the percent change in R_2_ between the “Off” and “On” states for each model. The hexamer again stands out, showing a 52.1% increase, which closely mirrors the 58.1% R_2_ increase observed experimentally during the glutamate sensing in MRS studies. This strong agreement between the simulation and experimental data suggests that, under glutamate-rich conditions, CA-SPIONs may predominantly form six-membered, equidistant aggregates, leading to a substantial contrast enhancement in T_2_-weighted MRS. These simulation results offer mechanistic insight into the behavior of the CA-SPION-based glutamate sensor. The clear relationship between the aggregation size and R_2_ contrast, along with the alignment between the simulated and experimental data, reinforces the conclusion that glutamate-induced clustering activates the sensor by enhancing the T_2_ contrast. This understanding may aid in the rational design of future magnetic nanosensors with tunable sensitivity and optimized response characteristics for in vitro molecular neuroimaging applications [62].

## 4. Conclusions

In summary, this study demonstrates the promise of citric acid-coated superparamagnetic iron oxide nanoparticles as effective glutamate-responsive contrast agents for magnetic resonance spectroscopy. These ultrasmall (5 nm) CA-SPIONs exhibited substantial T_2_ signal changes specifically in response to a range of physiologically relevant glutamate concentrations, with a significant relaxivity change ratio of 58.1% at 10.0 mM glutamate. In contrast, minimal responses were observed in the presence of GABA and dopamine, underscoring the selectivity of the CA-SPIONs toward glutamate. The underlying sensing mechanism, glutamate-induced nanoparticle aggregation, was further validated through computational modeling, which aligned well with experimental results and provided insight into the contrast enhancement via the cluster formation. The colloidal stability and responsiveness of these iron-based CA-SPION nanoprobes position them as eco-friendly alternatives to conventional contrast agents. Importantly, this work introduces a promising platform for the efficient in vitro monitoring of glutamatergic activity, which could support the diagnosis and study of neurodegenerative and neuropsychiatric conditions such as Alzheimer’s disease, epilepsy, and stroke. Future research will focus on evaluating the CA-SPION performance in cell-based systems and progressing toward in vivo imaging, laying the foundation for advanced molecular imaging tools in neuroscience.

## Data Availability

Data is contained within the article or Supplementary Materials.

## Supplementary Materials

The following supporting information can be downloaded at: https://www.mdpi.com/article/10.3390/s25144326/s1, Figure S1. The hydrodynamic diameter distributions of CA-SPIONs without and with neurotransmitters measured by dynamic light scattering spectroscopy, showing a significant aggregation of CA-SPIONs in 10 mM glutamate, while mainly being inert to other neurotransmitter conditions. Figure S2. Magnetic resonance spectroscopy (MRS) of CA-SPIONs at a wide range of dopamine concentrations: 0 (dark), 1.0 (red), 10.0 (light blue), and 10.0 (light green) μM. The x-axis is time in seconds, and the y-axis is the relative MR signal intensity. The transversal relaxation rates remained nearly unaltered, proving that CA-SPIONs are insensitive primarily to dopamine in a broad concentration window. Table S1. The calculated r_2_ values of CA-SPIONs under different dopamine concentrations at 1.9 Tesla. Figure S3. (a) MRS of CA-SPIONs at 1.9 Tesla in the absence of neurotransmitters, in which the x-axis represents the iron concentrations of CA-SPION samples in mM and the y-axis represents the R_1_ relaxation rates in s^−1^. The linear fit model yields a baseline r_1_ value of 1.94 mM^−1^s^−1^ at 1.9 Tesla. (b) MRS of CA-SPIONs at varied glutamate concentrations: 0 (dark), 4.0 (red), 7.0 (light blue), 10.0 (light green), 13.0 (purple), and 16.0 (yellow-green) mM. The x-axis is time in seconds, and the y-axis is the relative MR signal intensity. The longitudinal relaxation rates mainly remained unchanged, indicating that CA-SPION aggregation exerts minimal influence on T_1_ contrast. Table S2. The calculated r_1_ values of CA-SPIONs under different glutamate concentrations at 1.9 Tesla.
