# Supplementary material for: Ultrasmall Superparamagnetic Magnetite Nanoparticles as Glutamate-Responsive Magnetic Resonance Sensors
Source: Sensors (Basel). 2025 Jul 10;25(14):4326. doi: 10.3390/s25144326 (PMC12298936; doi:10.3390/s25144326)
Supplement: Supplementary file 1 [file sensors-25-04326-s001.zip › sensors-3704110-supplementary.pdf]

Supporting Information

Ultrasmall Superparamagnetic Magnetite Nanoparticles as Glutamate-responsive Magnetic Resonance Sensors

Hannah Mettee [1], Aaron Asparin [1], Zulaikha Ali [1], Shi He [2], Xianzhi Li [1], Joshua Hall [1], Alexis Kim [1], Shuo Wu [2,*], Morgan J. Hawker [1], Masaki Uchida [1], and He Wei [1,*]

1 *Department of Chemistry and Biochemistry, California State University Fresno, 2555 E San Ramon Ave, Fresno, CA 93740, USA*
2 *Department of Electrical and Computer Engineering, California State University Fresno, 2320 E San Ramon Ave, Fresno, CA 93740, USA*

*: corresponding authors are hewei@mail.fresnostate.edu (H.W., primary) and shuowu@mail.fresnostate.edu (S.W.)

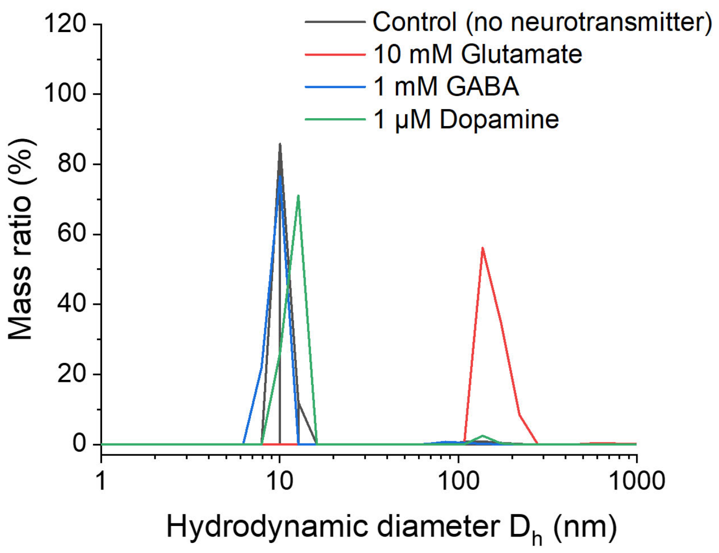

Figure S1. The hydrodynamic diameter distributions of CA-SPIONs without and with neurotransmitters measured by dynamic light scattering spectroscopy, showing a significant aggregation of CA-SPIONs in 10 mM glutamate, while mainly being inert to other neurotransmitter conditions.

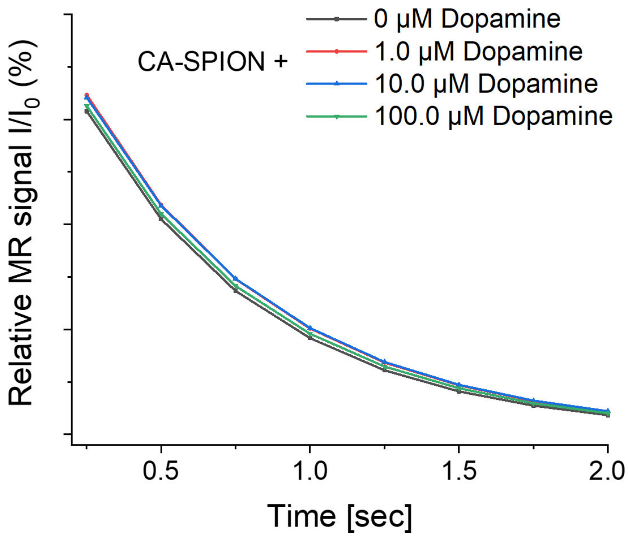

Figure S2. Magnetic resonance spectroscopy (MRS) of CA-SPIONs at a wide range of dopamine concentrations: 0 (dark), 1.0 (red), 10.0 (light blue), and 10.0 (light green) μM. The x-axis is time in seconds, and the y-axis is the relative MR signal intensity. The transversal relaxation rates remained nearly unaltered, proving that CA-SPIONs are insensitive primarily to dopamine in a broad concentration window.

Table S1. The calculated r_2 values of CA-SPIONs under different dopamine concentrations at 1.9 Tesla.

Dopamine concentration (μM)	r_2 ($mM^{-1}s^{-1}$)
**0**	3.66 ± 0.01
**1.0**	3.40 ± 0.07
**10.0**	3.48 ± 0.14
**100.0**	3.58 ± 0.09

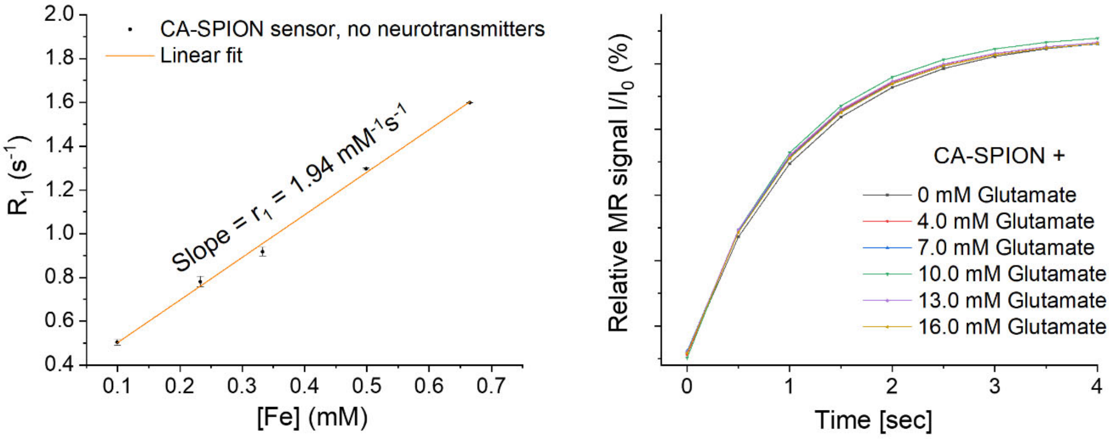

Figure S3. (a) MRS of CA-SPIONs at 1.9 Tesla in the absence of neurotransmitters, in which the x-axis represents the iron concentrations of CA-SPION samples in mM and the y-axis represents the R_1 relaxation rates in s^{-1}. The linear fit model yields a baseline r_1 value of 1.94 $mM^{-1}s^{-1}$ at 1.9 Tesla. b) MRS of CA-SPIONs at varied glutamate concentrations: 0 (dark), 4.0 (red), 7.0 (light blue), 10.0 (light green), 13.0 (purple), and 16.0 (yellow-green) mM. The x-axis is time in seconds, and the y-axis is the relative MR signal intensity. The longitudinal relaxation rates mainly remained unchanged, indicating that CA-SPION aggregation exerts minimal influence on T_1 contrast.

Table S2. The calculated r_1 values of CA-SPIONs under different glutamate concentrations at 1.9 Tesla.

**Glutamate concentration (mM)**	**r_1 ($mM^{-1}s^{-1}$)**
**0**	1.94 ± 0.06
**4.0**	2.01 ± 0.07
**7.0**	1.96 ± 0.02
**10.0**	2.11 ± 0.12
**13.0**	2.00 ± 0.02
**16.0**	1.93 ± 0.02
**GABA concentration (mM)**	r_1 ($mM^{-1}s^{-1}$)
**1.0**	1.94 ± 0.01
**Dopamine concentration (µM)**	r_1 ($mM^{-1}s^{-1}$)
**1.0**	1.83 ± 0.08
**10.0**	1.83 ± 0.14
**100.0**	1.91 ± 0.09
